# Haploidentical related donor vs matched sibling donor allogeneic hematopoietic stem cell transplantation for acute myeloid leukemia and myelodysplastic syndrome aged over 50 years: A single‐center retrospective study

**DOI:** 10.1002/cam4.3290

**Published:** 2020-07-20

**Authors:** Jiafu Huang, Fen Huang, Zhiping Fan, Na Xu, Li Xuan, Hui Liu, Pengcheng Shi, Ling Jiang, Yu Zhang, Jing Sun, Qifa Liu

**Affiliations:** ^1^ Department of Hematology Nanfang Hospital Southern Medical University Guangzhou China

**Keywords:** acute myeloid leukemia, allogeneic hematopoietic stem cell transplantation, elderly, haploidentical related donor, matched sibling donor, myelodysplastic syndrome

## Abstract

Allogeneic hematopoietic stem cell transplantation (allo‐HSCT) is a potentially curative therapeutic option for patients with acute myeloid leukemia (AML) or myelodysplastic syndrome (MDS). Increasing data suggest that haploidentical donor (HID) transplantation achieve comparable outcomes with HLA‐matched sibling donor (MSD) in adult AML/MDS. This retrospective study compared the outcomes of AML or MDS patients age ≥50 years underwent HID and MSD transplantation. One hundred and fifty‐six patients were enrolled in this study, including 75 HID and 81 MSD transplantation. The 100‐day cumulative incidence of II‐IV° acute graft‐versus‐host disease (GVHD) was 33.3 ± 5.4% vs 22.2 ± 4.6%, respectively, in HID and MSD groups (*P* = .066), and III‐IV° acute GVHD was not significantly different between two groups (5.3%±2.6% vs 6.2%±2.7%, respectively, *P* = .823). The 2‐year cumulative incidence of limited and extensive chronic GVHD was not statistically different in HID and MSD groups (20.9 ± 5.5% vs 18.9 ± 4.8% and 13.0 ± 4.7% vs 19.7 ± 5.0%, *P* = .889 and *P* = .269, respectively). The 2‐year cumulative incidences of relapse (27.0 ± 5.6% vs 22.7 ± 5.1%, *P* = .509), 2‐year overall survival (63.0 ± 5.8% vs 66.7 ± 5.4%, *P* = .454), 2‐year transplant‐related mortality (17.2 ± 4.6% vs 17.4 ± 4.4%, *P* = .847), 2‐year progression‐free survival (59.3 ± 5.8% vs 64.5 ± 5.4%, *P* = .437), 2‐year GVHD‐free relapse‐free survival (42.6 ± 5.9% vs 40.9 ± 5.6%, *P* = .964) were not significantly different in the two groups. The present data showed equivalent outcomes in AML or MDS patients age ≥50 years underwent HID and MSD transplantation.

## Graphical abstract

We compared the transplant outcomes between HID and MSD transplants for AML and MSD aged ≥50 years. The results showed that HID transplant is feasible and safe for elderly AML/MDS patients.

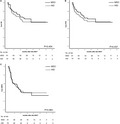

## 1 BACKGROUND

Allogeneic hematopoietic stem cell transplantation (allo‐HSCT) is a curative therapy for patients with acute myeloid leukemia (AML) or myelodysplastic syndrome（MDS）. However, most of these patients are older adults aged ≥60 years.^1^ Historically, older adults were not taken into account in allo‐HSCT given frequent comorbidities and higher transplant‐related mortality. With technical advance in allo‐HSCT, it has been broadened the application of older population, and its upper age limit has risen from 40 to 45 to 70 and to 75 years over the past four decades.^2^, ^3^ A growing number of studies have demonstrated that allo‐HSCT results in improvement of outcomes and is not a contraindication for older AML and MDS.^4^, ^5^, ^6^, ^7^, ^8^

Although HLA‐identical sibling donor (MSD) is the best choice for allo‐HSCT, it is difficult to older patients since siblings would be expected to be similar age and often unavailable or ineligible.^9^ In comparison, haploidentical donor (HID) is available to nearly all patients requiring allo‐HSCT. Over the last decade, the efficacy and safety of HID transplants in hematologic malignancies have been confirmed. Some studies showed that HID might achieve comparable outcomes with HLA‐matched sibling donor (MSD) in hematologic malignancies.^10^, ^11^, ^12^, ^13^ In this report, we compared the transplant outcomes between HID and MSD transplants for AML and MSD aged ≥50 years. The present data showed equivalent outcomes in AML or MDS patients age ≥50 years underwent HID and MSD transplantation.

## 2 METHODS

### 2.1 Study design and data collection

This is a retrospective study based on the transplantation database in our center. The inclusion criteria of this retrospective study were patients aged ≥50 years diagnosed with de novo AML or MDS who underwent HID transplant or MSD transplant between January 2013 and December 2018. The genetic risk of AML was based on the European Leukemia Network (ELN) 2017 recommendations and the cytogenetics risk of MDS was based on the Revised International Prognostic Scoring System (IPSS‐R).^14^, ^15^ This study was performed in accordance with the principles of the Declaration of Helsinki. Data were obtained from the patients’ medical records. Variables collected for all patients included demographic features, pretransplant‐related parameters, transplant‐related parameters, and graft‐versus‐host disease (GVHD), relapse‐related parameters, treatment‐related parameters, survival, infections, and so on. Written informed consent for submitting data to our database was routinely obtained when a patient was admitted to our center.

### 2.2 HLA typing

High‐resolution DNA typing for HLA‐A, HLA‐B, HLA‐C, HLA‐DRB1, and HLA‐DQB1 was performed for all patients and donors. MSDs were related sibling donors matching ≥9/10 HLA and HIDs were related donors matching 5‐8/10 HLA.^16^ Donor‐specific anti‐HLA antibody (DSA) screening was performed for HID, donors were ineligible if DSA were found.^17^ MSD was the first choice for allo‐HSCT. If MSD was unavailable, if patients did not have a suitable HLA‐matched unrelated donor, or if a patient's disease status left insufficient time for an unrelated donor search, patients were considered for HID HSCT.^18^

### 2.3 Conditioning and Transplants

All patients received myeloablation conditioning regimens including BuCy (busulfan 3.2 mg kg^−1^ d^−1^, days −7 to −4; cyclophosphamide 60 mg kg^−1^ d^−1^, days −3 and −2; and simustine 250 mg/m^2^, day −3) or BF (busulfan 3.2 mg kg^−1^ d^−1^, days −6 to −3; fludarabine 30 mg/m^−2^/d^−1^, days −7 and −3; and simustine 250 mg/m^2^, day −3) or TBI + Cy (total body irradiation 4.5 Gy/d, days −5 and −4; cyclophosphamide 60 mg kg^−1^ d^−1^, days −3 and −2). All HID patients were transplanted with a combination of bone marrow (BM) and peripheral blood stem cell (PBSC) grafts, whereas all MSD patients received PBSC grafts. Cyclosporin A (CsA), methotrexate (MTX) (on days +1, +3, and +6), and mycophenolate (MMF) were administered to patients undergoing MSD transplant for GVHD prophylaxis. CsA + MTX + MMF + ATG (total dose, 7.5 mg/kg on days −3 to −1 or 10 mg/kg on days −4 to −1) was administered to patients undergoing HID transplant for GVHD prophylaxis.^19^, ^20^

### 2.4 Evaluation points and definitions

This study mainly focused on engraftment, GVHD, relapse, transplant‐related mortality (TRM), overall survival (OS), progression‐free survival (PFS), GVHD‐free relapse‐free survival (GRFS). Assessments of engraftment were previously described in detail.^21^ Relapse was defined by morphologic evidence in the peripheral blood, marrow, or extramedullary sites. TRM was estimated as death without evidence of leukemia recurrence. PFS was defined as survival in continuous complete remission without hematological relapse. GRFS was defined as the absence of III‐IV° aGVHD, cGVHD requiring systemic therapy, relapse, or death.^22^ aGVHD and cGVHD were graded according to the literature.^23^

### 2.5 Statistical analysis

Our study data were analyzed on May 15, 2020. Comparisons of categorical variables were made by means of chi‐squared and Fisher exact tests for small numbers. Differences between numerical variables were calculated by means of two‐sample t test. Incidence of time‐dependent variables was estimated by the method of Kaplan‐Meier. The Cox’ regression model was used for analyzing prognostic factors for relapse, PFS, TRM, and OS. Numerical variables were analyzed as categories based on their values being below or above the median of the entire cohort. All statistical tests were two‐sided, and *P*‐value less than .05 was considered statistically significant. A multivariate analysis was performed using Cox proportional hazards model. Variables were included in the multivariate model if they were conceptually important or if they approached or attained statistical significance by univariate analysis. All data analysis was performed on the SPSS 24.0(SPSS, IBM, USA).

## 3 RESULTS

### 3.1 Patient clinical and transplants characteristics

A total of 156 AML or MDS patients aged ≥50 years after allo‐HSCT were enrolled in this retrospective study, including 75 HID and 81 MSD. The median age of the patients was 58.0 (range, 50.4‐69.0) years in HID group and 57.5 (range, 50.5‐68.0) in MSD group (*P* = .741). The median follow‐up was 25.2 m (range, 0.4‐73.3 m) in the HID group and 27.9 m (range, 2.1‐74.3 m) in the MSD group (*P* = .409). Sixty patients in the HID group were diagnosed as AML (50 CR and 10 no‐CR) and 15 were MDS. In MSD group, 63 patients were AML (52 CR and 11 no‐CR) and 18 were MDS, respectively. The proportion of patients with refractory AML of the two groups was similar (*P* = .999). In HID group, 40 patients received BuCy, 23 patients received BF, and 12 patients received TBI + Cy regimens. In the MSD group, 41 patients received BuCy, 29 patients received BF, and 11 patients received TBI + Cy regimens. Characteristics of patients, donors, and transplants are summarized in Table 1. Significant differences were noted in the donors’ age, stem cell source, and the family relationship of recipients and donors between both groups. There were no significant differences in patients’ age, gender, gender match, disease status, cytogenetics/molecular genetics risk, conditioning regimen, hematopoietic cell transplantation comorbidity index(HCT‐CI), time of follow‐up, doses of nucleated cells between the two groups. Patients’ clinical and transplant characteristics are shown in Table 1.

**TABLE 1:** Patient clinical and transplants characteristics

Characteristics	HID group (N = 75)	MSD group (N = 81)	*P* value
Age, median (range)	58.0 (50.4‐69.0)	57.5 (50.5‐68.0)	.741
Sex			.999
Male	51 (68.00%)	56 (69.14%)	
Female	24 (32.00%)	25 (30.86%)	
Follow‐up in months, median (range)	25.2 (0.4‐73.3)	27.9 (2.1‐74.3)	.409
Disease, N (%)			.845
AML	60 (80.0%)	63 (77.8%)	
MDS	15 (20.0%)	18 (22.2%)	
Stem cell source N (%)			<.0001
BM + PBSC	75 (100%)		
PBSC		81 (100%)	
AML in CR, N (%)			.999
CR	50 (83.3%)	52 (82.5%)	
No CR	10 (16.7%)	11 (17.5%)	
Genetics risk of AML, N(%)			.858
Favorable risk	6 (10.0%)	6 (9.5%)	
Intermediate risk	8 (13.3%)	9 (14.3%)	
Poor risk	28 (46.7%)	25 (39.7%)	
Not available	18 (30.0%)	23 (36.5%)	
Cytogenetics risk of MDS, N(%)			.719
Very good	0	0	
Good	0	0	
Intermediate	4 (26.7%)	2 (11.1%)	
Poor	2 (13.3%)	3 (16.7%)	
Very poor	3 (20.0%)	4 (22.2%)	
Not available	6 (40.0%)	9 (50.0%)	
Donor age, median (range)	26 (14‐49)	49 (37‐63)	<.0001
Sex mismatch, N (%)			.169
Female donor/Male recipient	19 (25.3%)	29 (35.8%)	
Others(M/M,F/F,M/F)	56 (74.7%)	52 (64.2%)	
Relationship between donor and recipient, N (%)			<.0001
Sibling	20 (26.7%)	81 (100%)	
Child	55 (73.3%)	0	
HCT‐CI score			
0‐2	61 (81.3%)	68 (84.0%)	.678
≥3	14 (18.7%)	13 (16.0%)	
Mononucleated cell count (range, 108/kg)	9.9 (3.5‐13.5)	10.0 (5.9‐38.0)	.442

Abbreviations: AML, acute myeloid leukemia; BM, bone marrow; CR, complete remission; HCT‐CI, hematopoietic cell transplantation comorbidity index; HID, haploidentical related donor; MDS, myelodysplastic syndrome; MSD, matched sibling donor; PBSC, peripheral blood stem cell.

### 3.2 Engraftment

All patients achieved hematopoietic reconstitution except one patient in the HID group who died of graft failure. Neutrophils reconstruction occurred in the HID group at a median of 12 d (range, 9‐18) and in the MSD group at a median of 12 d (range, 8‐22), respectively (*P* = .458). Platelet reconstruction in the HID and MSD groups occurred at a median of 13 d (range, 10‐53) and 13 d (range, 8‐91), respectively (*P* = .333).

### 3.3 GVHD

**FIGURE 1:**
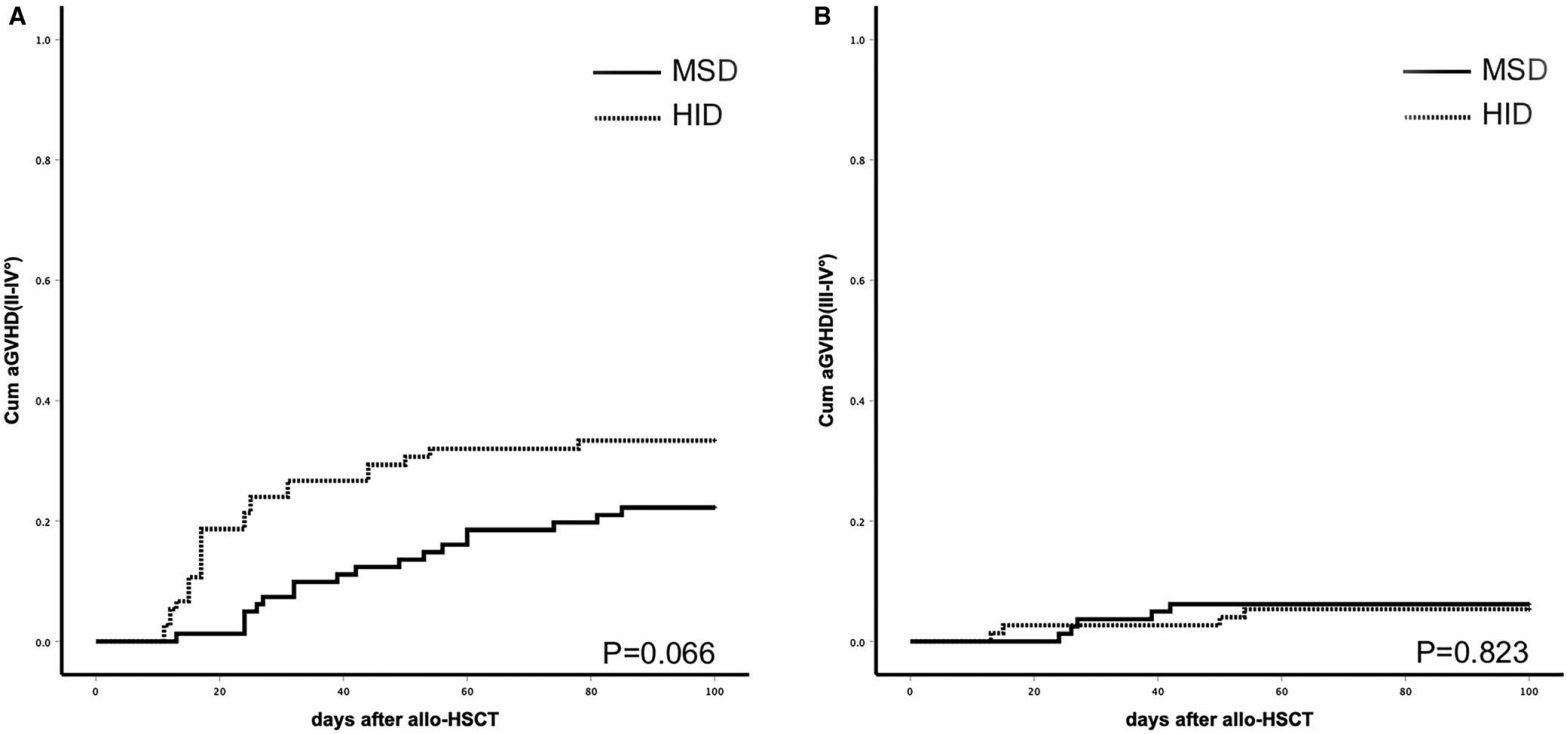
A, Cumulative incidence of II‐IV°aGVHD after HID or MSD transplants (*P* = .066); (B) cumulative incidence of III‐IV° aGVHD (*P* = .823)

**FIGURE 2:**
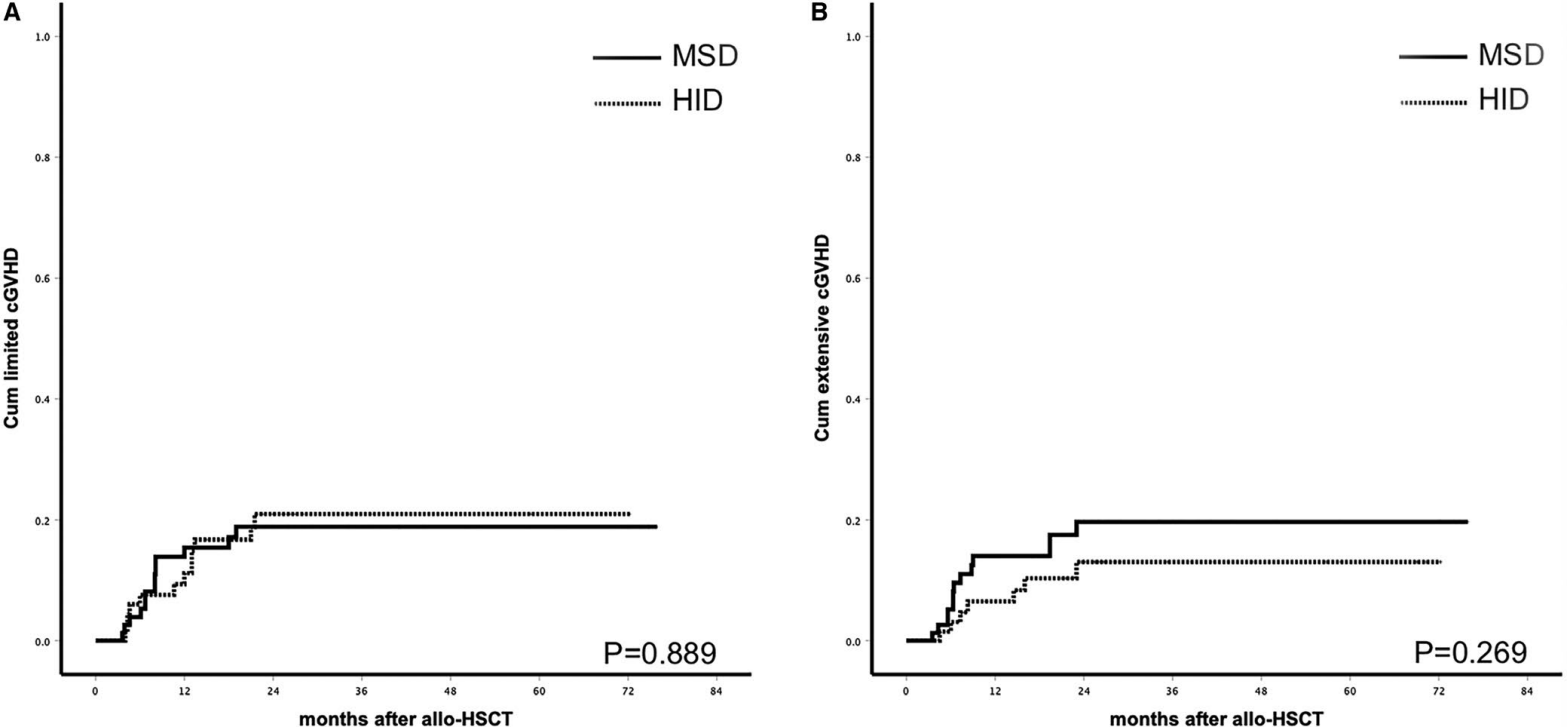
A, Cumulative incidence of limited cGVHD(*P* = .889);(B) cumulative incidence of extensive cGVHD(*P* = .269)

The 100‐day cumulative incidence of II‐IV° aGVHD were 33.3% ± 5.4% vs 22.2% ± 4.6%, respectively, in HID and MSD groups (*P* = .066). Incidence of III‐IV° aGVHD was 5.3% ± 2.6% vs 6.2% ± 2.7%, respectively, in HID and MSD groups (*P* = .823). One patient died of IV° gut aGVHD in MSD group, while no patients died of aGVHD in HID group. The 2‐year cumulative incidence of limited and extensive cGVHD was 20.9% ± 5.5% vs 18.9% ± 4.8% and 13.0% ± 4.7% vs 19.7% ± 5.0%, respectively, in HID and MSD groups (*P* = .889 and *P* = .269, respectively). Two patients died of cGVHD in HID group, four died of cGVHD in MSD group (2.67% vs 4.94%, *P* = .683). Incidence of aGVHD and cGVHD are shown in Figure 1 and Figure 2. Sex mismatch was significantly associated with higher risk of II‐IV°aGVHD and cGVHD (HR 2.369 CI 1.118‐5.101 *P* = .024 and HR 1.901 CI 1.314‐4.893 *P* = .027) in multivariate analysis (Table 3).

### 3.4 TRM

**FIGURE 3:**
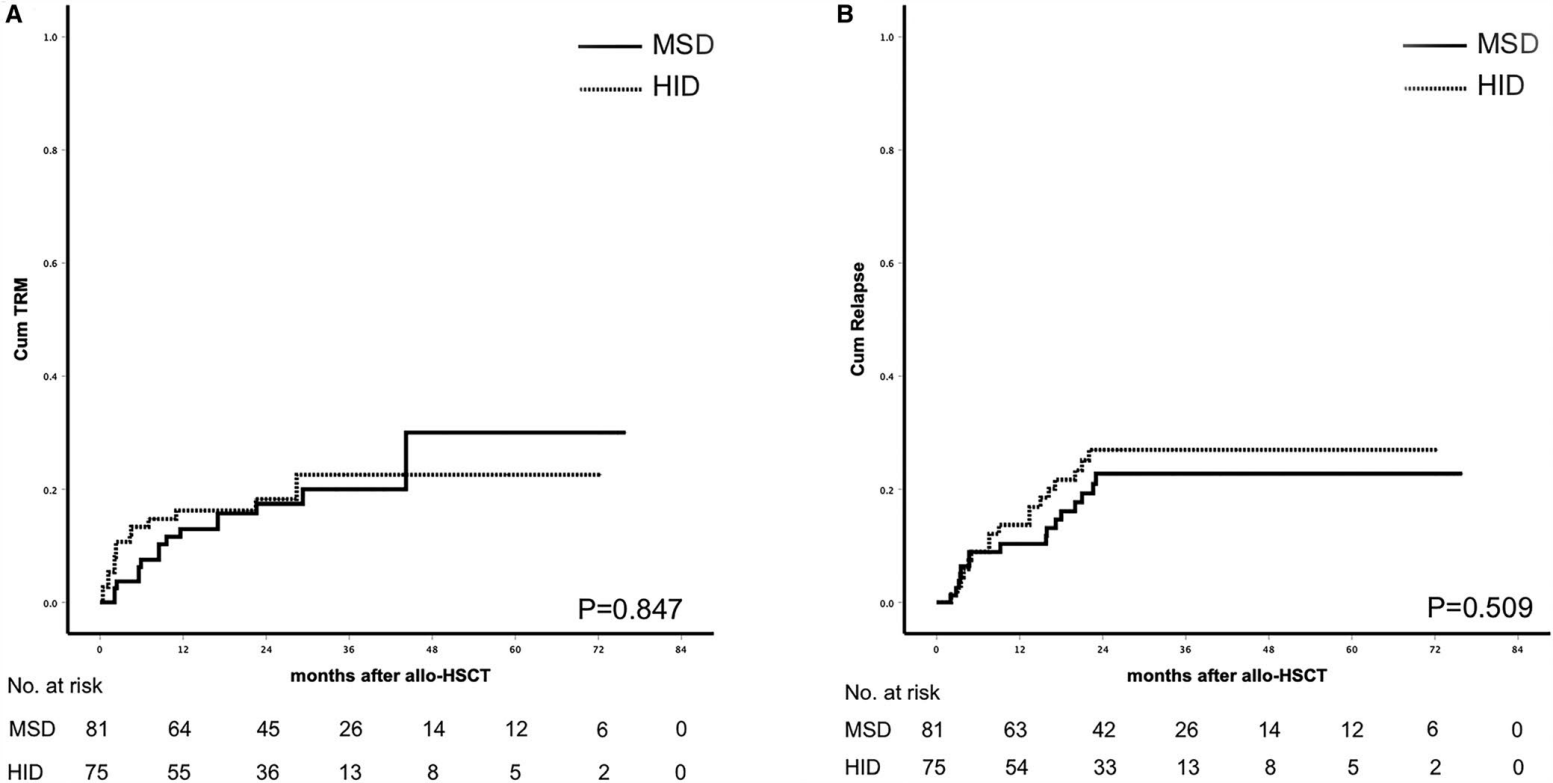
A, Cumulative incidence of TRM (*P* = .847); (B) cumulative incidence of relapse (*P* = .509)

The causes of death included relapse (n = 30) and TRM (n = 30). Of the 30 patients who died of TRM, infections (63.3%, n = 19) were the main cause, including nine (30.0%) infectious diseases for HID recipients, 10 (33.3%) for MSD recipients. Other causes included aGVHD (3.3%, n = 1), cGVHD (20.0%, n = 6), thrombotic microangiopathy (6.7%, n = 2), cerebral infarction (3.3%, n = 1), graft rejection (3.3%, n = 1). The 2‐year cumulative incidences of TRM in the HID and MSD groups were 17.2% ± 4.6% vs 17.4% ± 4.4% (*P* = .847, Figure 3A). Patient's age and sex mismatch were significantly associated with higher risk of TRM (HR 1.767 CI 1.040‐8.532 *P* = .021 and HR 2.843 CI 1.453‐7.142 *P* = .002) in multivariate analysis (Table 3).

### 3.5 Infections

Patients in the HID group has significantly higher rates of CMV DNAemia (*P* = .001). The incidence of other major infectious complications, including sepsis, CMV disease, EBV DNAemia, and invasive fungal infection, was not significantly between the two groups (Table 2).

**TABLE 2:** Characteristics of infectious complications post‐SCT

	HID group(N = 75)	MSD group(N = 81)	P
Sepsis	15 (20.0%)	12 (14.8%)	0.407
CMV DNAemia	48 (64.0%)	20 (24.7%)	0.001
CMV disease	3 (4.0%)	3 (3.7%)	0.999
EBV DNAemia	5 (6.7%)	4 (7.23%)	0.739
Invasive fungal infection	24 (32.0%)	27 (33.3%)	0.866
Urinary tract infection	6 (8.0%)	2 (2.5%)	0.155

Abbreviations: CMV, cytomegalovirus; EBV, Epstein‐Barr virus; HID, haploidentical related donor; MSD, matched sibling donor.

### 3.6 Relapse and survival

**FIGURE 4:**
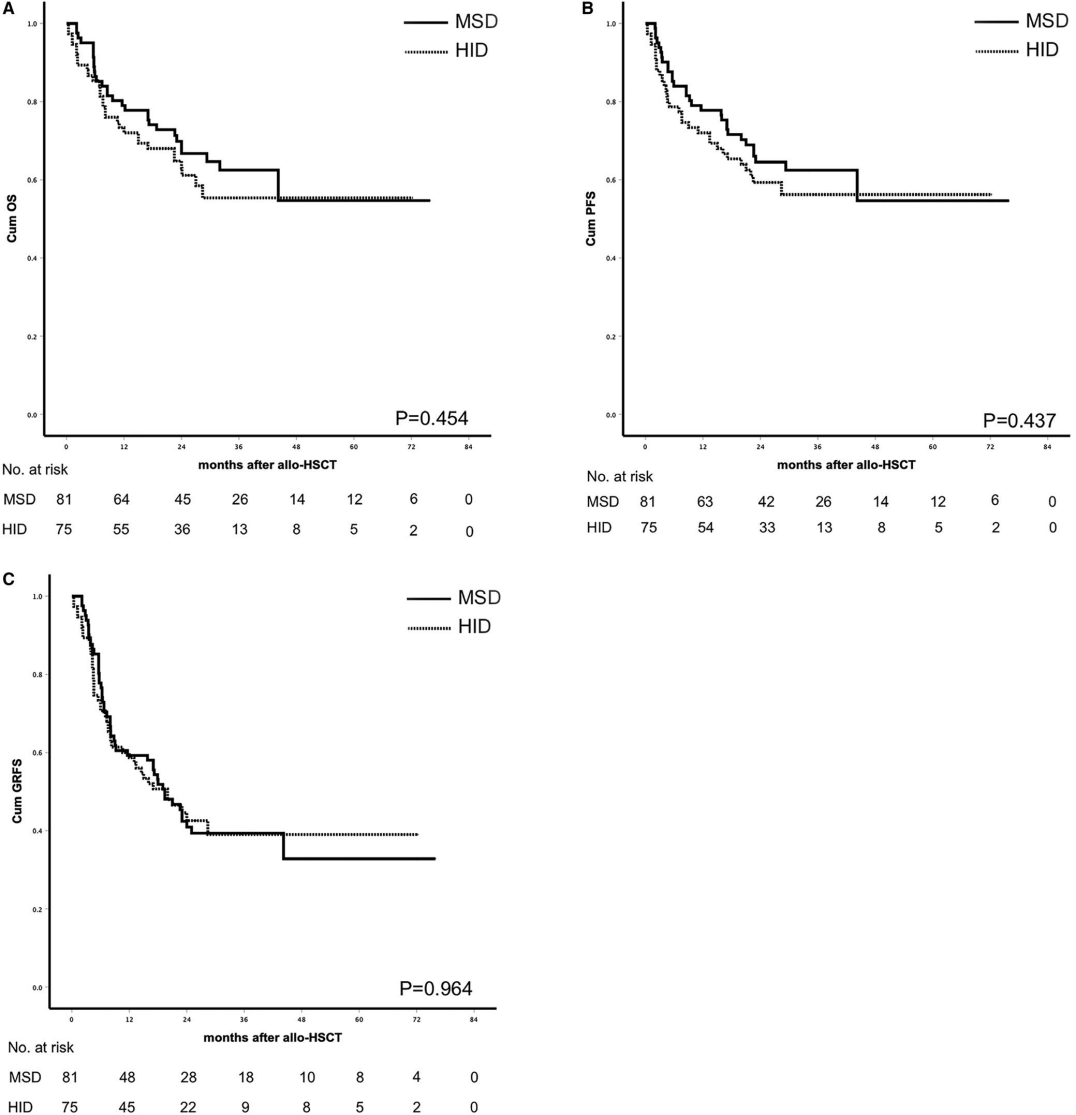
A, Probability of survival (*P* = .454); (B) probability of PFS (*P* = .437); (C) probability of GRFS (*P* = .964)

**FIGURE 5:**
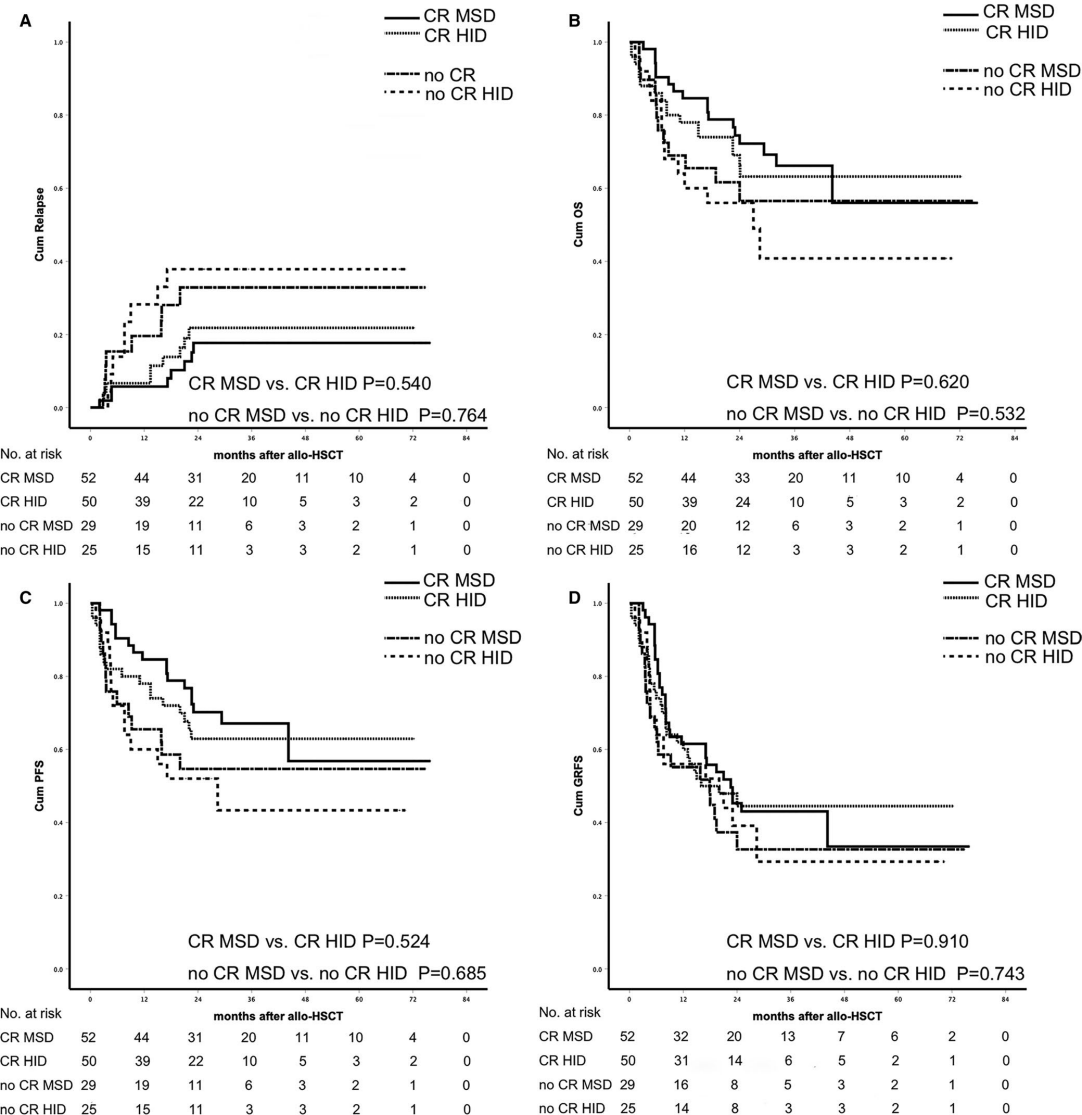
Subgroup analysis of relapse and survival. (A) cumulative incidence of relapse (CR MSD vs CR HID *P* = .540, no CR MSD vs no CR HID *P* = .764). (B) probability of survival (CR MSD vs CR HID *P* = .620, no CR MSD vs no CR HID *P* = .532); (C) probability of PFS (CR MSD vs CR HID *P* = .524, no CR MSD vs no CR HID *P* = .685); (D) probability of GRFS (CR MSD vs CR HID *P* = .910, no CR MSD vs no CR HID *P* = .743)

The median follow‐up was 25.2 m (0.4‐73.3) in the HID group and 27.9 m (2.1‐74.3) in the MSD group (*P* = .409). No difference was observed in the cumulative incidence of relapse, PFS, OS, and GRFS according to donor type. Leukemia relapse occurred in 17 and 16 patients, respectively, in HID and MSD group (*P* = .601). The 2‐year cumulative incidence of relapse (27.0% ± 5.6% vs 22.7% ± 5.1%, *P* = .509), 2‐year PFS (59.3% ± 5.8% vs 64.5% ± 5.4%, *P* = .437), 2‐year OS (63.0% ± 5.8% vs 66.7% ± 5.4%, *P* = .454), and 2‐year GRFS(42.6% ± 5.9% vs 40.9 ± 5.6%, *P* = .964) was not significantly different in the HID and MSD groups (Figure 3B, Figure 4). Disease status at transplants was significantly associated with higher risk of relapse (HR 6.121 CI 2.275‐17.635 *P* < .001) in multivariate analysis (Table 3). Patient's age (older than 60), disease status at transplants, and sex mismatch were independent risk factors for OS (HR 1.526 CI 1.014‐3.607 *P* = .043, HR 3.261 CI 1.639‐6.811 *P* = .002, and HR 2.447 CI 1.342‐6.212 *P* = .001) and PFS (HR 1.611 CI 1.112‐3.504 *P* = .048, HR 3.584 CI 1.621‐7.904 *P* = .001, and HR 2.721 CI 1.644‐6.721 *P* = .001). Of the CR patients (50 in HID group and 52 in MSD group), the 2‐year cumulative incidence of relapse (21.8% ± 6.5% vs 17.7% ± 5.7%, *P* = .540), 2‐year PFS (62.9% ± 7.0% vs 70.2% ± 6.5%, *P* = .524), 2‐year OS (66.2% ± 7.0% vs 72.2% ± 6.4%, *P* = .620), and 2‐year GRFS (44.5% ± 7.4% vs 45.3 ± 7.0%, *P* = .910) were not significantly different in the HID and MSD groups. Of the no CR patients (25 in HID group vs 29 in MSD group), the 2‐year cumulative incidence of relapse (37.8% ± 10.6% vs 32.9% ± 9.6%, *P* = .764), 2‐year PFS (52.0% ± 10.0% vs 54.7% ± 9.3%, *P* = .685), 2‐year OS (56.0% ± 9.9% vs 56.5% ± 9.7%, *P* = .532), and 2‐year GRFS (39.1% ± 10.0% vs 32.7% ± 9.1%, *P* = .743) was not significantly different in the HID and MSD groups (Figure 5). The outcomes of CR or no CR patients received HID and MSD transplantation were not significantly different.

**TABLE 3:** Multivariate analysis of outcomes

Outcome/variable	HR(95% CI)	*P*
OS		
Patients age		
≥60	1.526 (1.014‐3.607)	**.043**
<60	1	
Disease status at SCT		
No CR	3.261 (1.639‐6.811)	**.002**
CR	1	
Sex mismatch		
Female donor/male recipient	2.447 (1.342‐6.212)	**.001**
Others(M/M,F/F,M/F)	1	
Type of transplant		
HID	1.023 (0.732‐1.871)	.262
MSD	1	
PFS		
Patients age		
≥60	1.611 (1.112‐3.504)	**.048**
<60	1	
Disease status at SCT		
No CR	3.584 (1.621‐7.904)	**.001**
CR	1	
Sex mismatch		
Female donor/male recipient	2.721 (1.644‐6.721)	**.001**
Others(M/M,F/F,M/F)	1	
Type of transplant		
HID	1.019 (0.682‐1.879)	.324
MSD	1	
TRM		
Patients age		
≥60	1.767 (1.040‐8.532)	**.021**
<60	1	
Disease status at SCT		
No CR	1.541 (0.411‐6.731)	.601
CR	1	
Sex mismatch		
Female donor/male recipient	2.843 (1.453‐7.142)	**.002**
Others(M/M,F/F,M/F)	1	
Type of transplant		
HID	1.156 (0.479‐1.762)	.401
MSD	1	
Relapse		
Patients age		
≥60	1.314 (0.391‐4.765)	.729
<60	1	
Disease status at SCT		
No CR	6.121 (2.275‐17.635)	**<.001**
CR	1	
Sex mismatch		
Female donor/male recipient	2.194 (0.706‐6.233)	.159
Others(M/M,F/F,M/F)	1	
Type of transplant		
HID	0.971 (0.503‐1.811)	.323
MSD	1	
II‐IV°aGVHD		
Patients age		
≥60	1.216 (0.427‐3.453)	.601
<60	1	
Sex mismatch		
Female donor/male recipient	2.369 (1.118‐5.101)	.024
Others(M/M,F/F,M/F)	1	
Type of transplant		
HID	1.342 (0.602‐2.517)	.185
MSD	1	
cGVHD		
Patients age		
≥60	1.236 (0.510‐2.981)	.688
<60	1	
Sex mismatch		
Female donor/male recipient	1.901 (1.314‐4.893)	.027
Others(M/M,F/F,M/F)	1	
Type of transplant		
HID	1.201 (0.621‐3.032)	.471
MSD	1	

Abbreviations: aGVHD, acute graft‐versus‐host disease; cGVHD, chronic graft‐versus‐host disease; CR, complete remission; HID, haploidentical related donor; MSD, matched sibling dono; OS, overall survival; PFS, progression‐free survival; TRM, transplant‐related mortality.

## 4 DISCUSSION

Here, we report the outcomes of HID vs MSD transplants for patients with AML/MDS aged ≥50 years. The results showed that the two cohorts had comparable outcomes including TRM, GVHD, relapse, and survival.

Traditionally, allo‐HSCT in the elderly has a higher TRM because of the patient's frequent comorbidities and poor performance status. Reports from main transplant centers for older patients have shown 2‐year TRM rates ranging from 7% to 35%.^2^, ^7^, ^24^, ^25^, ^26^ Some studied reported that myeloablative conditioning regimen (MAC) was associated with higher TRM rates compared to those who underwent nonmyeloablative conditioning (NMAC) or reduced intensity (RIC) conditioning regimen.^27^, ^28^, ^29^, ^30^ The report from Seattle of 1055 patients undergoing allo‐HSCT showed that the 2‐year TRM was 14%, 21%, and 41% for the patients with HCT‐CI scores 0, 1‐2, and 3 or more, respectively.^31^ Beelen et al reported 476 older or comorbid AML/MDS patients with a median HCT‐CI of 3.0 undergoing allo‐HSCT showed that the 2‐year TRM was up to 28.2%.^32^ In the present study, the 2‐year cumulative incidences of TRM were 17.2% in the HID and 17.4% MSD in the HID. A reasonable interpretation of the relatively lower TRM is that our patients have relatively lower HCT‐CT. In this report, only 14 (18.7%) of the patients in HID group and 13 (16.0%) of the patients in MSD group have HCT‐CI scores ≥3. Besides, 23 patients (30.7%) in HID group and 29 patients (35.8%) in MSD group received BuF MAC. This may also be one of the reasons for the lower TRM. Many researchers have shown that patients received BuF MAC has a lower TRM incidence than that of received BuCy.^33^, ^34^ Whether HID transplants have a higher TRM than MSD is currently under discussion. A growing number of studies show that there is no difference between HID and MSD in TRM, including the elderly.^24^, ^25^, ^26^, ^35^, ^36^ Similar results were obtained in this study.

Relapse is a major cause of failure in patients undergoing allo‐HSCT. Many factors influence relapse, such as donor resources, disease status at transplants, patient's age, conditioning, and so on.^37^, ^38^, ^39^, ^40^ For donor resources, some studies showed that HID had stronger GVL than MSD transplantation, making relapse lower.^41^, ^42^, ^43^ Other studies suggested that there was no difference in the relapse rate between the two donor sources.^13^, ^24^, ^25^, ^44^, ^45^ In the present study, there was no difference in relapse between two groups. In multivariate analysis, our result showed that disease status at transplant was independent risk factor for relapse. This result was consistent with other studies.^40^, ^46^, ^47^, ^48^ Ogawa et al retrospectively analyzed the data of AML patients registered in the Japan Society of Hematopoietic Stem Cell transplantation who underwent allo‐SCT and were and confirmed that survival of patients with relapsed or refractory AML was poor due to the increased relapse.^40^ Ikegame et al reported the result of a multicenter phase I/II study of HID allo‐HSCT, the non‐CR status at transplantation was the significant prognostic factor of increased relapse, which tended to be associated with a lower survival.^48^

GVHD is the most common transplant‐related complication that affects the outcomes of transplants.^49^, ^50^ Over the last decade, great improvements have been made in prophylaxis for GVHD in HID transplantation, especially the use of T‐cell depletion in vivo by means of Cy or ATG.^24^, ^42^, ^51^ The Cy‐based HID transplantation was associated with higher relapse rate, which was up to 50% while ATG‐based HID has showed superiority in reducing relapse.^52^, ^53^ A major concern related to ATG‐based HID transplantation was the high TRM rate. Studies have shown that the TRM of ATG‐based HID was higher than that of the Cy‐based HID.^54^ However, Tang et al reported the comparison of ATG‐ and Cy‐based HID, TRM in the ATG group was lower than that in the Cy group.^55^ Choose between the two HID platforms remains controversial. Traditionally, HID was associated with higher incidence of GVHD compared with MSD transplantation. A growing number of evidences showed that the incidences of GVHD in HID were not different from that in MSD, especially cGVHD.^16^, ^24^, ^35^, ^41^, ^51^, ^56^ In the ATG protocol, previous results showed that the incidences of grade II‐IV° aGVHD were higher for HID than MSD, but the incidences of severe aGVHD and cGVHD were comparable between two groups.^16^, ^41^, ^42^ In this study, HID was associated with a trend of higher incidences of grade II‐IV° aGVHD than MSD. In multivariate analysis, we found that sex mismatched donor (female donor/male recipient) was significantly associated with higher risk of aGVHD and cGVHD. Several other studies have also reported the same results.^57^, ^58^, ^59^, ^60^, ^61^

GRFS reflects the main complications of allo‐HCT and represents the real recovery following allo‐HCT.^22^ Previous study showed that GRFS of HID transplant was comparable to that of MSD.^62^ Mehta et al reported HID offered the best GRFS compared to other alternative donors in a retrospective analysis.^63^ Studies compared GRFS between MSD and HID transplants in elderly AML/MDS patients were limited. In the present study, HID and MSD achieved very similar GRFS.

As we know, ATG as GVHD prophylaxis was associated with higher incidences of infections, especially fatal viral infections.^64^, ^65^, ^66^ In the present study, although HID was associated with much higher incidences of CMV‐anemia than MSD, the number of deaths caused by viral diseases and other infectious diseases did not differ between two groups. A possible explanation for these results might be attributed to the extensive experience at the study centers in effectively managing infectious diseases, resulting in many patients with CMV or other infections avoiding TRM.

The limitations of this study are the relatively small number of patients and the nature of this retrospective single‐center study. We were not able to perform subgroup analyses that would have been informative in some specific setting such as advanced cytogenetics and/or older age. Sample size limitations of the oldest age group may mask smaller differences in outcomes. Selection bias may also have influenced inferences from the data. It is possible that the older patients included in these transplants were a highly selected group with a lower score of HCT‐CI.

## 5 CONCLUSION

The present data showed similar outcomes in patients aged 50 years and older underwent HID compared to MSD at our institution. We conclude that HID transplant is feasible and safe for elderly AML/MDS patients. The lack of an HLA‐identical donor in elderly patients with AML/MDS should not preclude allo‐SCT.

## Data Availability

The raw/processed data required to reproduce these findings cannot be shared at this time as the data also forms part of an ongoing study.
